# Role of systemic corticosteroids in ventilated patients with chronic obstructive pulmonary disease exacerbation: a systematic review and meta-analysis

**DOI:** 10.1186/s12890-026-04327-7

**Published:** 2026-05-18

**Authors:** Tanvi Tiwari, Eduardo Saadi Neto, Frederico Augusto Travi Squizzato, Felix Reyes

**Affiliations:** 1 Department of Hospital Medicine, Meritus Medical Center, Hagerstown, Maryland USA; 2 Medical resident, Department of Emergency Medicine, Mayo Clinic, Rochester, Minnesota USA; 3 Medical student, Federal University of Bahia (Universidade Federal da Bahia) — UFBA, Salvador, Bahia Brazil; 4 Department of Pulmonary Medicine, Northwest Medical Center, Tucson, Arizona USA

**Keywords:** Acute exacerbation of chronic obstructive pulmonary disease, Respiratory failure, Systemic steroids, Mechanical ventilation, Non-invasive ventilation, Intensive care unit

## Abstract

**Purpose:**

The recommendation regarding systemic corticosteroids in ventilated patients with acute exacerbation of chronic obstructive pulmonary disease (AECOPD) is unknown since most studies have excluded ventilated patients. Our study aims to investigate whether systemic steroids benefit the subgroup of AECOPD who require ventilation.

**Methods:**

We systematically searched PubMed, Cochrane, and Embase databases for randomized trials or observational studies comparing systemic steroids to placebo or no systemic steroids, up to April 2025. Outcomes of interest were intensive care unit (ICU) mortality, length of ICU stay, duration of ventilation, non-invasive ventilation (NIV) failure rate, hyperglycemia episodes, and ventilator-associated pneumonia (VAP).

**Results:**

Six studies including 1596 ventilated AECOPD patients (including subgroups from larger ICU populations), were included in the systematic review. Of these 1424 contributed to the quantitative synthesis of at least one outcome. We performed sensitivity analysis for study type and mode of ventilation. ICU mortality was not significantly decreased in the systemic steroid group compared to placebo or no systemic steroids (Odds ratio (OR) = 1.09; 95% CI 0.62–1.91; *p* = 0.78; I²=0.0%). There was no statistically significant difference between the groups for duration of ventilation (Mean difference (MD) = -2.24; 95% CI -5.13 to 0.66; *p* = 0.13; I²=78.7%). In the subgroup of randomized controlled trials (RCTs), systemic steroids were associated with a shorter duration of ventilation (MD = -1.12 days; 95% CI -2.23 to 0.00; *p* = 0.049; I² = 59.9%). There was no difference between groups for length of ICU stay (MD = -1.60; 95% CI -3.32 to 0.12; *p* = 0.068; I²=68.5%). There was no statistically significant difference between groups for NIV failure (Odds ratio (OR) = 1.17; 95% CI 0.83–1.67; *p* = 0.37; I²=58.1%). Hyperglycemia episodes were significantly higher in the steroid group (OR = 2.38; 95% CI 1.56 to 3.62; *p* < 0.001; I²=0.0%). There was no significant difference in rates of VAP (OR = 1.19; 95% CI 0.80–1.77; *p* = 0.38; I²=0.0%).

**Conclusion:**

This meta-analysis suggests that critically ill AECOPD patients on ventilation may not derive a significant benefit from systemic steroids.

**Prospero protocol No:**

CRD420251034592.

**Supplementary Information:**

The online version contains supplementary material available at 10.1186/s12890-026-04327-7.

## Introduction

Chronic obstructive pulmonary disease (COPD) is a common, preventable and treatable disease, characterized by persistent respiratory symptoms and irreversible airflow limitation due to chronic airway inflammation from exposure to noxious particles or gases [1]. In 2023 COPD was the fifth leading cause of death in the United States resulting in 141,733 deaths annually [2].

Acute exacerbation of COPD (AECOPD) is defined as acute worsening of respiratory symptoms that result in additional therapy [3, 4]. Exacerbations contribute substantially to disease progression, accelerated lung function decline, and increased mortality risk [5]. The updated 2025 Global Strategy for the Diagnosis, Management, and Prevention of COPD (GOLD) report recommends systemic corticosteroids for hospitalized AECOPD patients or those with significant exacerbations (evidence Grade A), but cautions that even short bursts of corticosteroids can cause significant adverse effects, warranting use only in significant events [4].

The current recommendation stems from evidence demonstrating that systemic corticosteroids shorten recovery time, improve forced expiratory volume in 1 s (FEV1), improve oxygenation, decrease risk of early relapse, treatment failure, and length of hospitalization [6]. However, most of these trials excluded critically ill patients, leaving uncertainty regarding the efficacy and safety of corticosteroids in critically ill patients, especially those requiring ventilation.

A 2014 meta-analysis by Abroug et al. evaluated corticosteroids versus placebo or standard therapy in AECOPD, including a subgroup of ICU patients (*n* = 300). While corticosteroids improved overall treatment success, no benefit was observed in the critically ill subgroup, regardless of ventilation mode, and adverse effects were more frequent [7]. The authors concluded that evidence was insufficient to recommend systemic corticosteroids in ventilated AECOPD and called for targeted trials in this population [7].

Since then, two cohort studies and one RCT have addressed this question, with conflicting results. To address this gap, we conducted an updated systematic review and meta-analysis including both RCTs and observational studies of AECOPD patients, requiring mechanical ventilation (MV) or non-invasive ventilation (NIV), comparing outcomes between systemic steroids and placebo or no steroids.

## Methods

### Study design

This systematic review and meta-analysis was registered in the International Prospective Register of Systematic Reviews (PROSPERO) under protocol number CRD420251034592. This review was conducted following the Preferred Reporting Items for Systematic Reviews and Meta-analyses (PRISMA) guidelines [8] and in accordance with the Cochrane Collaboration Handbook for Systematic Review of Interventions [9]. The present review utilized the PICOT (Participants, Intervention, Comparison, Outcomes, Type of study) framework: Population (P): Patients suffering from acute exacerbation of COPD on ventilatory support; Intervention (I): use of systemic corticosteroids; Comparison (C): Placebo or no use of systemic corticosteroids; Outcomes (O): (1) Primary: ICU mortality, length of ICU stay, duration of ventilation and NIV failure rate (2) Secondary: hyperglycemia episodes, ventilator-associated pneumonia(VAP); type of study (T): randomized controlled trials and observational studies.

### Search strategy

A comprehensive search strategy was developed and executed across three electronic databases - PubMed, Embase, and Cochrane from inception to April 2025. The search strategy included keywords and MESH terms such as “chronic obstructive pulmonary disease,” “chronic obstructive airway disease”, “chronic obstructive lung disease”, “artificial ventilation”, “mechanical ventilation,” “Non-invasive ventilation,” “systemic corticosteroids”, “steroid”, “methylprednisolone”, “prednisone”, “dexamethasone,” “corticosteroid”. The literature search was restricted to English language studies only. The references from all included studies, previous systematic reviews and meta-analyses were also searched manually for any additional studies. A detailed search strategy is provided in the supplementary material.

### Study selection

All references retrieved through the database search were screened independently by two reviewers (T.T. and E.S.N.) using Rayyan© software [10]. The screening process was blinded between reviewers until conclusion. Full texts were then assessed for final inclusion. Disagreements between reviewers were resolved through discussion, and when consensus could not be reached, the senior author (F.R.) adjudicated.

### Eligibility or inclusion criteria

We included RCTs and observational studies comparing systemic steroids to placebo or no systemic steroids in patients with COPD exacerbation on ventilation, either mechanical (MV) or non-invasive ventilation (NIV). Any studies with our population of interest as a subgroup that reported at least one outcome of interest were included. There were no restrictions related to publication year. Exclusion criteria included articles not available in full text, studies that excluded ventilated AECOPD patients, preprints, letters to the editor, conference proceedings, case reports and series, reviews, gray literature, and studies with insufficient data on relevant outcomes.

#### Duration of ventilation

Some studies such as Alia et al., Bahloul et al., Abdalah et al. and Abroug et al., reported raw ventilation duration for the whole cohort of ventilated patients or defined it as a sum of NIV and MV support. Galerneau et al. and Alia et al., reported ventilation duration for MV and NIV subgroups separately. Alia et al. and Abroug et al. also defined criteria for weaning, SBT failure, and the weaning process.

#### NIV failure

NIV failure was defined in some studies; however, criteria differed. Galerneau and colleagues defined NIV failure as death under NIV or need for MV. Abroug et al., defined it as treatment failure requiring MV (GCS ≤ 8 and PaCO2 ≥ 60 mmHg, pH ≤ 7.2, cardiac arrest, PaO2 < 45 mmHg despite maximum tolerate FiO2). Alia et al., defined NIV failure as indication for intubation if any of the criteria met (7.20; pH 7.20–7.25 on 2 measures 1 h apart; hypercapnic coma with GCS < 8 and PaCO2 ≥ 60 mm Hg; PaO2 < 45 mm Hg despite maximum tolerated FiO2; and/or cardiac arrest).

#### Hyperglycemia

Hyperglycemia thresholds differed between studies. Galerneau et al., defined hyperglycemia as fasting blood glucose level > 11 mmol/L, Abroug et al. defined it as blood glucose ≥ 180 mg/dL in patients without preexisting diabetes requiring initiation of insulin or increase in existing insulin therapy. Bahloul et al., defined it as glucose ≥ 8 mmol/L while Alia et al., defined it as blood glucose ≥ 120 mg/dL in patients without pre-existing diabetes, or increased insulin dose in patients with diabetes.

### Data collection process

After screening, two reviewers (T.T. and F.T.S.) independently extracted data in duplicate using a standardized spreadsheet. Extracted data included study design, population size, demographic characteristics, details of the intervention and comparator, methodological features, and clinical outcomes. We preferentially extracted adjusted data for one of the cohort studies that performed propensity score matching. Any discrepancies were resolved through discussion, and if consensus could not be reached, a third reviewer (senior author F.R.) adjudicated.

### Risk of Bias

The risk of bias in individual randomized controlled trials was assessed using version 2 of the Cochrane Risk of Bias tool (RoB 2.0) [11]. The non-randomized studies were assessed using the Newcastle-Ottawa Scale, and the assessments were performed independently by two reviewers (T.T. and F.R.) [12]. Any disagreements were resolved through discussion between the two reviewers.

### Data analysis

Outcome definitions were reviewed across studies for similarity before pooling. Outcomes judged to be sufficiently similar in clinical practice were synthesized together. The differences were considered during interpretation of pooled estimates and acknowledged as a potential source of heterogeneity. Data was extracted for the subgroup of ventilated AECOPD where available and assessed for compatibility based on combinable effect measures. Studies were selected for inclusion in specific outcome analyses depending on the availability of subgroup data and similarity in definitions (T.T and F.T.S.). Studies were excluded from specific outcome analysis if they did not provide combinable outcome data exclusively for the ventilated subgroup. In one study, group-specific sample sizes were not reported for certain outcomes, and were derived from the total outcome specific sample size and event percentages, with rounding to the nearest whole number where necessary. Any disagreements were resolved by consensus, with arbitration by the senior author (F.R.) when necessary.

### Statistics

We calculated pooled effect sizes using the R© software version 4.5.0 and used a random effects model. We performed a sensitivity analysis for subgroups of mode of ventilation and type of study (RCT vs. observational study). Heterogeneity was assessed using I² statistics. Statistical significance was defined as a two-sided p value < 0.05. P-values were reported to two decimal places, with values < 0.001 reported as *p* < 0.001. We also performed leave-one-out sensitivity analyses for duration of ventilation and length of ICU stay to assess the influence of individual studies on the pooled estimates and between-study heterogeneity.

## Results

### Study selection and baseline characteristics

**Fig. 1:**
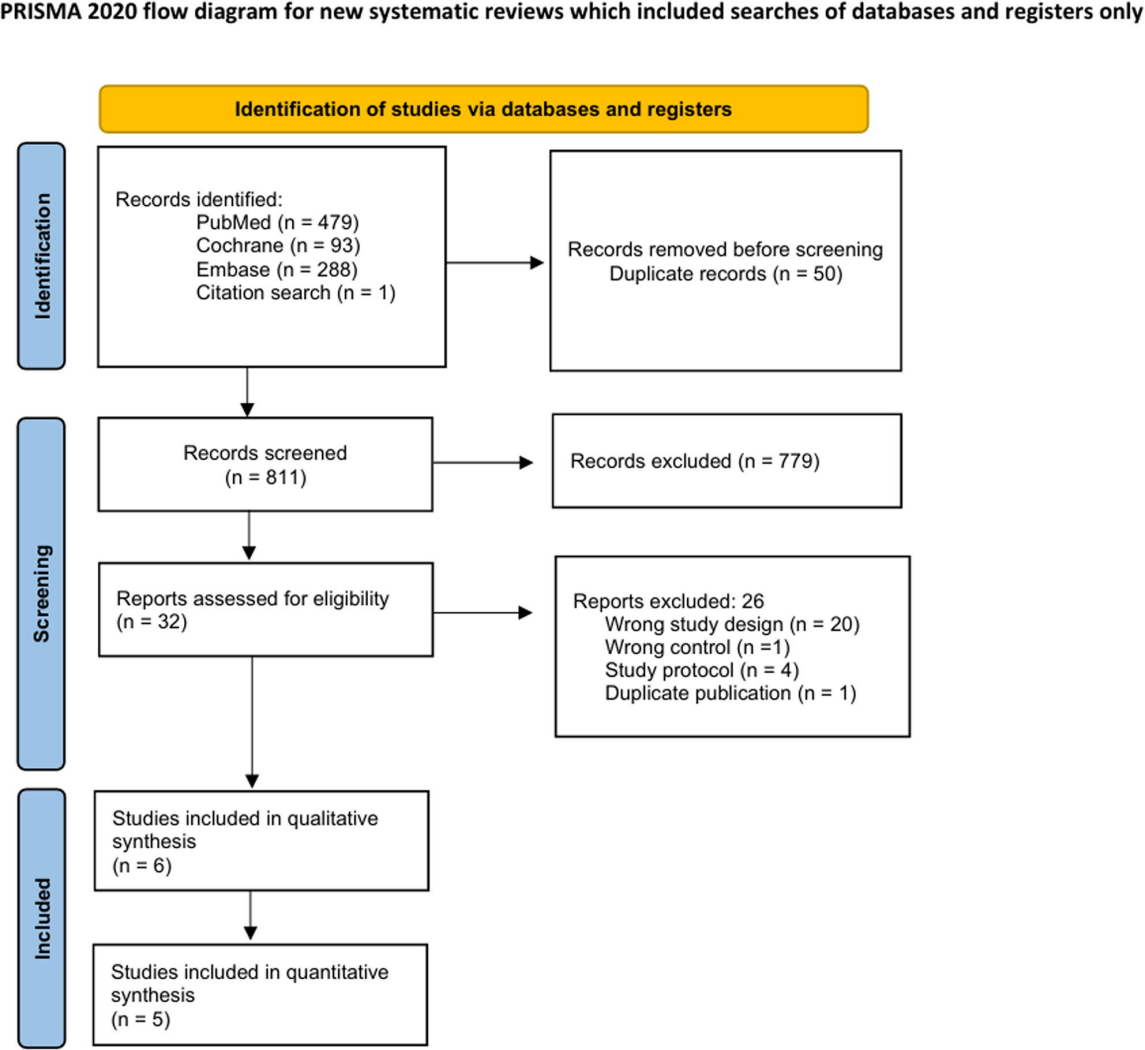
PRISMA 2020 flow diagram of study selection

The literature search identified 861 articles. Rayyan© software was used for deduplication. After deduplication, 811 articles were reviewed by title and abstract using Rayyan© software, of which 779 were excluded. The process was performed independently by two authors (T.T. and E.S.N.), and the results were blinded until screening completion. Thirty-two articles were reviewed in detail, of which six met the inclusion criteria for qualitative analysis. Five studies with a total of 1424 patients contributed to the quantitative synthesis. Any disagreements were resolved by consensus, with arbitration by the senior author (F.R.) where necessary. The selection process is illustrated in the PRISMA flow chart (Fig. 1).

### Characteristics of the studies

The population included predominantly males with a similar distribution between steroid and non-steroid groups, except for Bai et al., which performed propensity score matching with 50% males in both groups [13]. Most studies included older adults with mean ages across studies ranging from approximately 55 to 74 years. The proportion of patients with various comorbid conditions, such as congestive heart failure (CHF), hypertension, and type 2 diabetes mellitus, was similar between the steroid and non-steroid groups as well as between most studies. Abroug et al. had a relatively higher proportion of patients with concurrent cardiac decompensation [14]. The predominant cause of AECOPD was respiratory tract infection in most studies, and the proportion was similar between steroid and non-steroid groups. In one study by Bahloul et al., there was a larger proportion of patients with high simplified acute physiology (SAPSII) scores; 73% of patients were mechanically ventilated and had comorbid shock, indicating a relatively sicker set of patients [15]. Baseline pulmonary function parameters such as (FEV1% predicted and GOLD stage) were not reported in most studies except Abroug et al. (2014). While Galerneau et al. reported GOLD stage for only 290 of its 1247 cohort with pulmonary function tests (PFT). Illness severity in most studies was characterized using severity scores (SAPS2, APACHE2) and arterial blood gas values (PaCO2, pH and PaO2/FiO2).

### Summary of included studies

Six studies comprising of 1596 ventilated AECOPD patients were included in the systematic review. Among these, 977 received systemic corticosteroids, and 619 received placebo or no steroids. Initial ventilation was NIV in 868 patients and mechanical or invasive in 628 patients, with mode of ventilation not specified for 100 patients in Abdalah et al. [16]. Three studies were randomized trials: Abdalah et al. and Alia et al. were double-blind RCTs, whereas Abroug et al. was an open-label randomized controlled trial [14, 16, 17]. The remaining studies were observational, including propensity score matched cohort (Bai et al.), a retrospective 1:1 case-control study (Bahloul et al.), and a prospective observational cohort using inverse probability weighting (Galerneau et al.) [13, 15, 18].

Bai et al. and Galerneau et al. were mixed ICU cohorts with a subgroup of ventilated patients, whereas the remaining studies exclusively enrolled ventilated patients. Steroid type, duration, and timing varied between studies and are summarized in Table 1. Most studies reported outcomes including duration of ventilation (NIV and MV), ICU mortality, and ICU and hospital length of stay. Commonly reported complications were hyperglycemia, gastrointestinal bleeding, ventilator-associated pneumonia, hospital-acquired pneumonia. Bai et al. reported primary outcome of 28-day mortality in the ventilated subgroup; however, the other outcomes were not specified for the ventilated subgroup. Bai et al. also did not report any steroid complications [13].

**Table 1:** Systemic steroids used in the different studies

Study	Steroid	Dose	Duration
Abdalah et al. 2021 [16]	Methylprednisolone	0.5 mg/kg	10 days
Alia et al. 2011 [17]	Methylprednisolone	0.5 mg/kg	10 days
Bahloul et al. 2013 [15]	Hydrocortisone	100 mg	7 to 10 days
Abroug et al. 2014 [14]	Prednisone	1 mg/kg	Until discharge or 10 days
Bai et al. 2024 [13]	Prednisone-equivalent	> 0.5 mg/kg	At least 3 days
Galerneau et al. 2023 [18]	Prednisone-equivalent	≥ 0.5 mg/kg	NA

Galerneau et al. additionally assessed a composite outcome of death or invasive ventilation at day 28, survival at day 28 and 90, antibiotic use, NIV failure, and subgroup analyses by COPD severity, ventilation mode, and SAPS II score [18]. The criteria for COPD diagnosis, definition of exacerbation, physiologic thresholds for defining respiratory failure, and outcome definitions varied between studies. Some studies, like Abroug et al., defined COPD based on GOLD and spirometry criteria, while others, such as Galerneau et al. and Bai et al., included database/ICD-10 or clinician suspected diagnosis [14, 18]. Abdalah et al. did not provide any outcome definitions [16]. All study definitions are summarized in Table S2 in the supplementary material. The main clinical characteristics of the studies are summarized in Table 2.

### Outcome measures

The primary outcomes assessed are ICU mortality, length of ICU stay, duration of ventilation, and NIV failure rate. Secondary outcomes assessed are steroid complications of hyperglycemia and ventilator-associated pneumonia. Outcome definitions differed across studies and were reviewed for similarity before pooling. Outcomes judged to be sufficiently similar in terms of clinical practice were synthesized together. Some key outcome definitions are listed here. All definitions are summarized in Table S2 in the supplementary material.

**Table 2:** Baseline data of the included studies

Author, year of publication	Abdalah, 2021		Abroug, 2014		Alia, 2011		Bahloul, 2013		Bai, 2024		Galerneau, 2023	
Type of study	RCT		Open-label RCT		RCT		Pairwise case-control study		Cohort study		Cohort study	
Population	AECOPD requiring ventilatory support		AECOPD requiring ventilatory support		AECOPD requiring ventilatory support		AECOPD requiring ventilatory support		AECOPD admitted to ICU		AECOPD admitted to ICU	
	Steroids	No Steroids	Steroids	No Steroids	Steroids	No Steroids	Steroids	No Steroids	Steroids	No Steroids	Steroids	No Steroids
N of patients (ICU cohort)	50	50	111	106	43	40	17	17	206	206	391	856
Age (avg years ± SD)	55.11 ± 15	58.80 ± 20	N/A	N/A	69.1 (9.7)	67.6 (10.7)	70 ± 7.2	70 ± 11	73.7 ± 11.0	73.3 ± 10.9	N/A	N/A
Age (median [interquartile range])	N/A	N/A	70 [63–75]	68 [63–75]	N/A	N/A	N/A	N/A	N/A	N/A	71 [62–78]	70 [61–77]
Male n (%)	35 (70%)	37 (74%)	99 (89%)	92 (87%)	32 (74)	34 (85)	15	12	103 (50.0)	103 (50.0)	563 (65.8)	240 (61.4)
Hypertension n (%)	14 (28%)	12 (24%)	9 (8)	7 (7)	15 (35)	22 (55)	3	9	73 (35.4)	75 (36.4)	224 (26.2)	85 (21.74)
History of congestive HF n (%)	N/A	N/A	14 (13)	13 (12)	N/A	N/A	4	6	117 (56.8)	122 (59.2)	N/A	N/A
Diabetes mellitus n (%)	18 (36)	13 (26)	17 (15)	13 (12)	15 (35)	9 (22)	2	2	68 (33.0)	63 (30.6)	171 (20.0)	64 (16.37)
Respiratory tract infection n (%)	40 (80)	39 (78)	50 (45)	46 (43)	30 (70)	28 (70)	8	9	N/A	N/A	592 (69.2)	301 (77.0)
Sepsis n (%)	N/A	N/A	N/A	N/A	1 (2)	1 (2)	N/A	N/A	N/A	N/A	N/A	N/A
Cardiac n (%)	8 (16)	7 (14)	34 (31)	32 (30)	8 (19)	9 (22)	10	14	N/A	N/A	29 (3.4)	9 (2.3)
Postoperative n (%)	1 (2)	1 (2)	N/A	N/A	0 (0)	1 (2)	N/A	N/A	N/A	N/A	9 (1.1)	1 (0.3)
Unidentified n (%)	0	1 (2)	27 (24)	28 (27)	4 (9)	0 (0)	N/A	N/A	N/A	N/A	26 (3.0)	6 (1.5)
pH	7.32 ± 0.03	7.32 ± 0.02	7.28 (7.25–7.32)	7.29 (7.25–7.32)	7.27 ± 0.11	7.31 ± 0.10	7.31 ± 0.13	7.35 ± 0.10	7.3 ± 0.1	7.3 ± 0.1	7.34 [7.26;7.41]	7.31 [7.23;7.38]
PaCO2	66.64 ± 22.6	67.36 ± 21.65	78 (62–85)	80 (62–87)	69.9 ± 19.7	68.7 ± 18.5	56.3 ± 19.5	51 ± 20	55.2 ± 17.1	55.5 ± 17.5	57 [44; 73]	62 [47; 77]
PaO2/FiO2	1.83 ± 0.52	2.02 ± 0.56	N/A	N/A	197.8 ± 83.7	191.5 ± 75.9	255 ± 91	27 2 ± 121	N/A	N/A	N/A	N/A
NIV	N/A	N/A	84 (76)	80 (76)	18 (42)	19 (47)	3	4	62 (30.1)	63 (30.6)	174 (44.5)	361 (42.2)
MV	N/A	N/A	27 (24)	26 (24)	25 (58)	21 (52)	14	13	25 (12.1)	22 (10.7)	147 (37.6)	308 (36.0)
FEV1(mL/s)	N/A	N/A	820 (590–1120)	750 (565–950)	N/A	N/A	N/A	N/A	N/A	N/A	N/A	N/A
GOLD Stage III	N/A	N/A	33 (30)	29 (27)	N/A	N/A	N/A	N/A	N/A	N/A	133/290 (45.9)	
GOLD Stage IV	N/A	N/A	78 (70)	77 (73)	NA	NA	NA	NA	NA	NA	81/290 (27.9)	

*N/A* Unavailable data, *SD* Standard deviation, *PaCO2* Partial pressure of arterial carbon dioxide, *HF* Heart failure, *PaO2/FIO2* Partial pressure of arterial oxygen/fraction of inspired oxygen, *NIV* Non-invasive ventilation, *MV* Mechanical ventilation, *GOLD* Global strategy for diagnosis, management and prevention of chronic obstructive pulmonary disease, *FEV1 *Forced expiratory volume in 1 s

1: Population numbers and baseline characteristics for Bai et al. and Galerneau et al. represent their overall ICU cohorts, as isolated baseline data exclusively for the ventilated subgroups were not reported

2: Galerneau et al. reported outcome-specific N for certain outcomes, which differed from the subgroup population sizes

### Primary outcomes

#### ICU mortality

##### [4 studies; 434 patients]

**Fig. 2:**
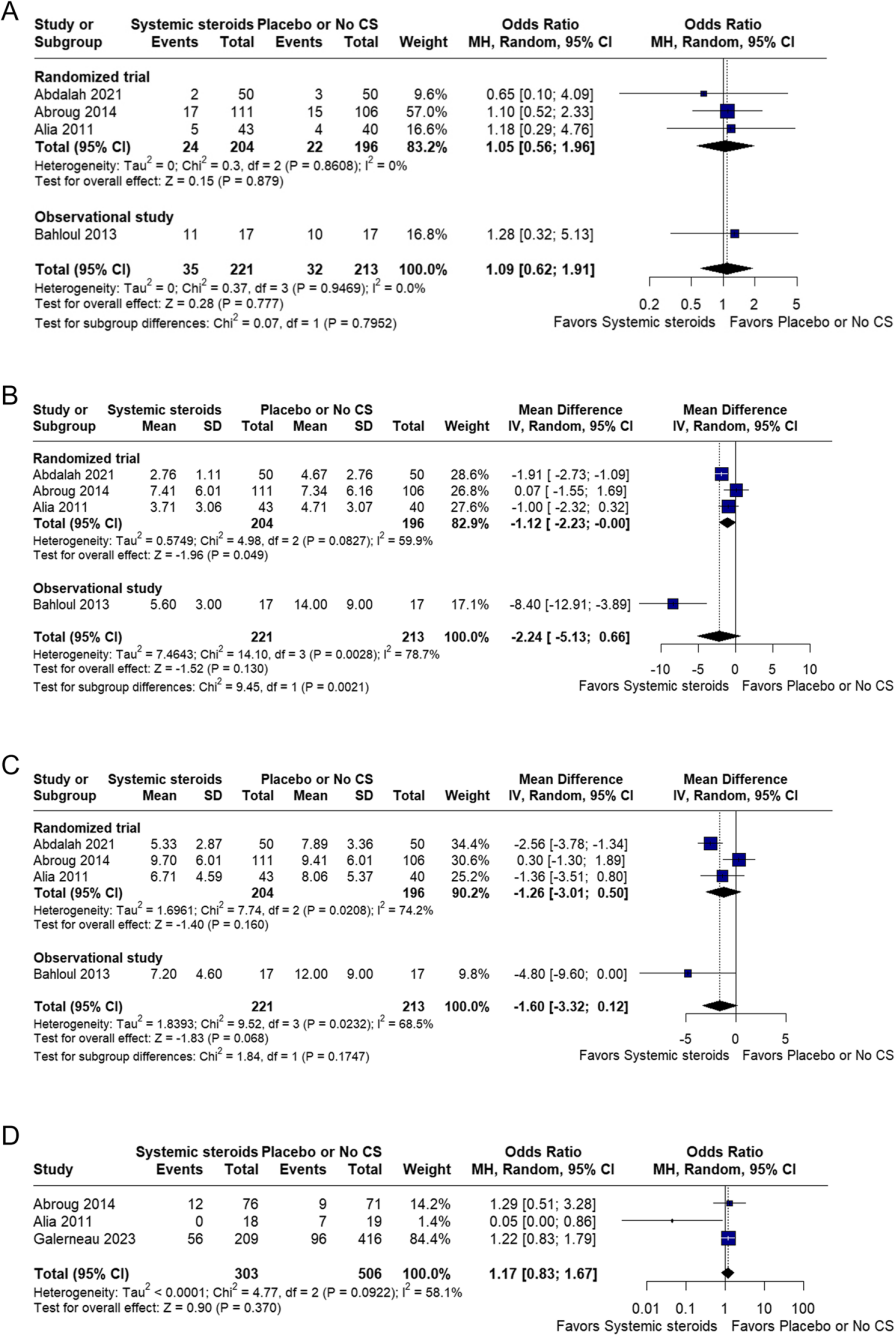
**A** Forest plot of ICU Mortality by study type (RCT and observational). **B** Forest plot of duration of ventilation by study type. **C** Forest plot of length of ICU stay by study type. **D** Forest plot of NIV failure rate

There was no statistically significant reduction in ICU mortality with corticosteroid therapy compared to control, with no between study heterogeneity. (OR = 1.09; 95% CI 0.62 to 1.91; *p* = 0.78; I² = 0.0%) (Fig. 2A). Results were consistent across RCT-only and observational subgroups.

#### Duration of ventilation

##### [4 studies; 434 patients]

Pooled analysis showed no statistically significant improvement in the duration of ventilation between corticosteroid therapy vs. control groups (MD = -2.24; 95% CI -5.13 to 0.66; p = 0.13; I² = 78.7%). In an RCT-only subgroup (*N* = 400), corticosteroid therapy decreased duration of ventilation by 1.1 days (MD = -1.12 days; 95% CI -2.23 to 0.00; p = 0.049; I² = 59.9%), with moderate heterogeneity (Fig. 2B).

#### Length of ICU stay

##### [4 studies; 434 patients]

Pooled analysis showed no statistically significant improvement in length of ICU stay with corticosteroid therapy, with substantial heterogeneity between studies (MD = -1.60; 95% CI -3.32 to 0.12; *p* = 0.07; I² = 68.5%). Results were consistent across RCT-only and observational study subgroups (Fig. 2C).

#### NIV failure rate

##### [3 studies; 809 patients]

Pooled analysis showed no statistically significant difference in NIV failure rate between steroid therapy and control groups, with moderate heterogeneity between studies (OR = 1.17; 95% CI 0.83 to 1.67; *p* = 0.37; I² = 58.1%) (Fig. 2D). For Galerneau et al., outcome-specific group sizes were derived from the reported event percentages; therefore, the denominator differs from the subgroup population in Table 2.

### Secondary outcomes

#### Hyperglycemia episodes

##### [4 studies; 434 patients]

**Fig. 3:**
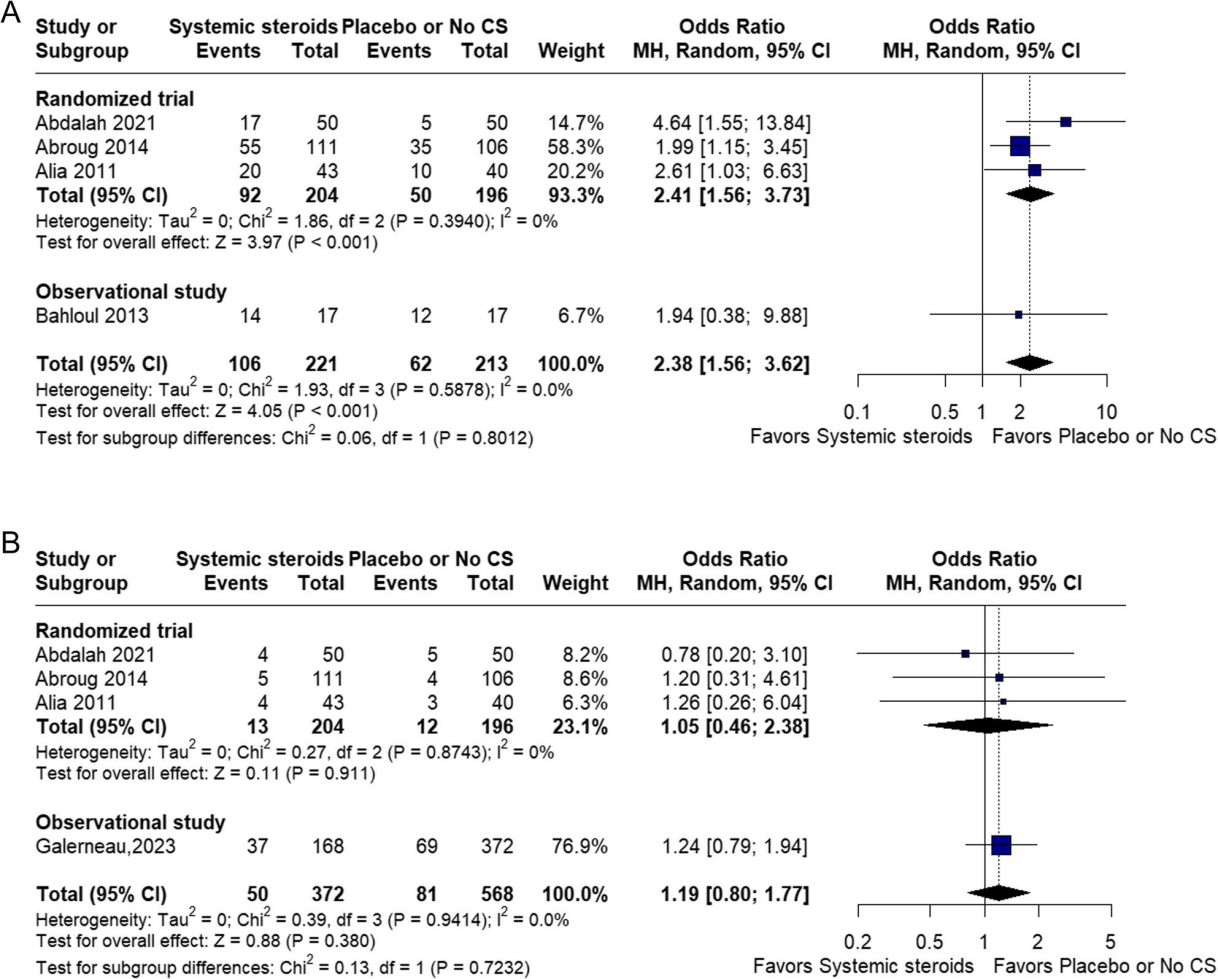
**A** Forest plot of hyperglycemia episodes by study type. **B** Forest plot of ventilator-associated pneumonia by study type

There was a statistically significant increase in hyperglycemia episodes in steroid therapy group compared to control, with no observed between study heterogeneity (OR = 2.38; 95% CI 1.56 to 3.62; *p* < 0.001, I² = 0.0%) (Fig. 3A).

#### Ventilator-associated pneumonia

##### [4 studies; 940 patients]

The pooled estimate showed no statistically significant difference in the incidence of VAP between steroid therapy vs. control groups (OR = 1.19; 95% CI 0.80 to 1.77; *p* = 0.38, I² = 0.0%) (Fig. 3B). For Galerneau et al., outcome-specific group sizes were derived from the reported event percentages; therefore, the denominator differs from the subgroup population in Table 2.

### Subgroup analyses

#### Duration of ventilation by mode of ventilation

**Fig. 4:**
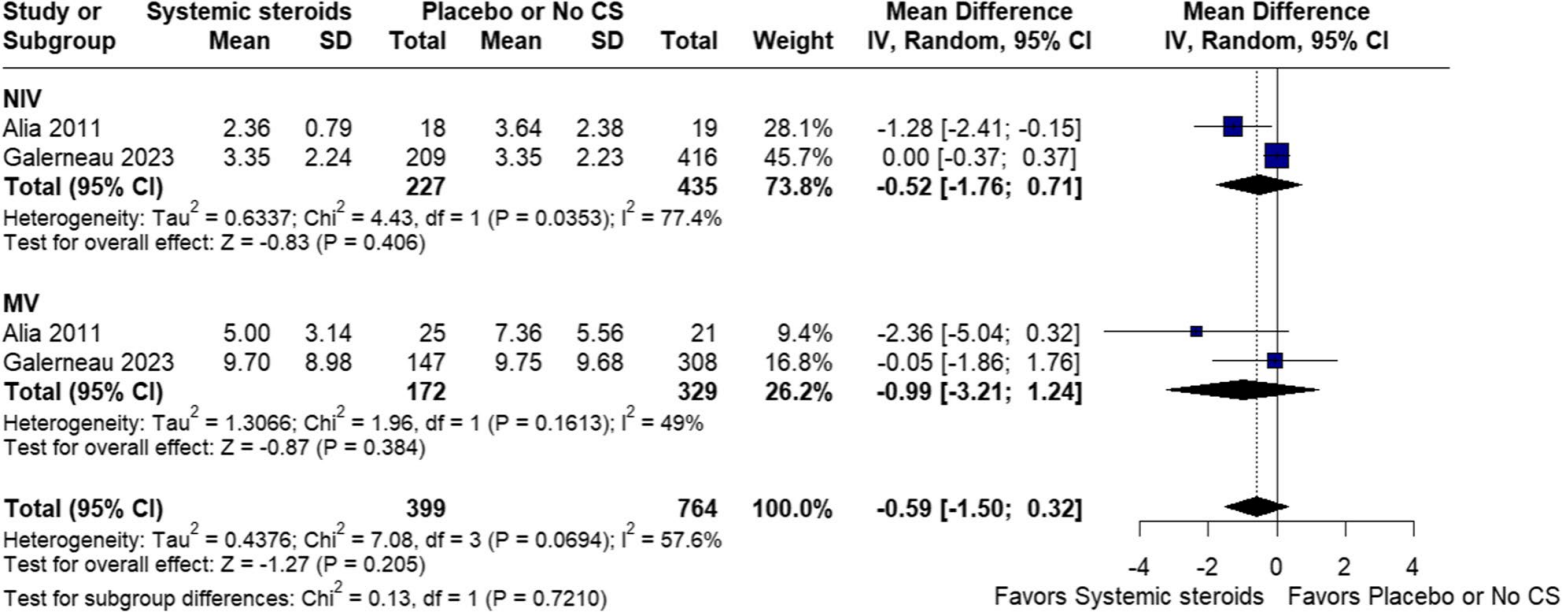
Forest plot of duration of ventilation by mode of ventilation (NIV and MV)

There was no statistically significant difference in duration of ventilation between steroid therapy and control groups, regardless of mode of ventilation (NIV, MD = -0.52; 95% CI -1.76 to 0.71; *p* = 0.41; I²=77.4%) and (MV, MD = -0.99; 95% CI -3.21 to 1.24; *p* = 0.38; I²=49.0%) (Fig. 4). Subgroup analysis showed moderate to high heterogeneity.

We also performed subgroup analysis for ICU Mortality by mode of ventilation: The pooled estimate showed no statistically significant difference between steroid therapy and control groups in either NIV or MV subgroups, with no heterogeneity between studies ( NIV, OR = 0.84; 95% CI 0.31 to 2.27; *p* = 0.74; I² = 0.0%; MV, OR = 1.42; 95% CI 0.57 to 3.54; *p* = 0.45, I² = 0.0%) (Figure S1). Bahloul et al. was excluded from this subgroup analysis as the study did not report ICU mortality outcome stratified by mode of ventilation.

### Sensitivity analysis

Leave-one-out analysis for the outcome of length of ICU stay, showed that omission of Abroug et al. resulted in a statistically significant reduction in length of ICU stay and markedly reduced heterogeneity (I² decreased from 68.5% to 0%), suggesting that this study contributed substantially to between-study heterogeneity and influenced the overall precision of the pooled estimate (Figure S2). The leave one out analysis for the outcome of duration of ventilation, demonstrated that exclusion of Bahloul et al. resulted in a statistically significant reduction in duration of ventilation and decreased heterogeneity (I² decreased from 78.7% to 59.9%), indicating that this study had a notable influence on heterogeneity and the overall pooled effect (Figure S3). Overall, no single study reversed the direction of the pooled estimates, but certain studies contributed to the observed heterogeneity, and results should therefore be interpreted with caution. The sensitivity analysis and leave-one-out analysis are presented in the supplementary materials.

### Risk of bias assessment

Risk of bias for randomized trials and observational studies is summarized in the supplementary Figure S4 and Table S1. Among the three RCTs, two were judged to be at low risk of bias and one had some concerns. The observational studies scored 5 to 6 stars on the Newcastle-Ottawa Scale, indicating moderate to high methodological quality.

## Discussion

An acute exacerbation of chronic obstructive pulmonary disease represents a trajectory-altering event in the lives of patients suffering from COPD, contributing to accelerated disease progression, increased healthcare utilization, and higher mortality rates, especially when ventilatory support is required [5]. This gradient of clinical deterioration from oxygen supplementation to NIV, and MV reflects a worsening trajectory of illness severity, reinforcing the substantial clinical and prognostic implications, and emphasizing the need for carefully optimized management [19]. Since systemic steroids can be associated with adverse effects, their use must be individualized and balanced against evidence-based benefits in this subset of critically ill AECOPD.

In this systematic review and meta-analysis of ventilated AECOPD patients, systemic steroids did not significantly improve ICU mortality, or NIV failure rate. Overall duration of ventilation and length of ICU stay showed an improvement in the steroid group compared to control. However, none of these results reached statistical significance. In the RCT-only subgroup, systemic steroids were associated with a 1.1-day shorter duration of ventilation; however, this finding was borderline and should be interpreted cautiously given the moderate heterogeneity. There was no significant difference in the rates of ventilator-associated pneumonia but a much higher rate of hyperglycemia in the steroid therapy group, which was statistically significant.

These findings are broadly consistent with the results of the previous meta-analysis by Abroug et al., and the recent studies by Galerneau et al. (2023) and Bai et al. (2024) [7, 13, 18]. Our study tried to expand on these findings through subgroup analysis by ventilation mode and study design. The consistent lack of benefit in certain outcomes suggests that once AECOPD progresses to respiratory failure, corticosteroids may offer little therapeutic advantage while exposing to potential steroid–related adverse effects.

Possible explanations include the advanced physiologic derangement and multimorbidity of these patients, as well as steroid related complications such as delirium, infections, hyperglycemia, critical illness myopathy which may outweigh anti-inflammatory benefits [20].

Although systemic steroids remain guideline-endorsed, the current evidence in ventilated patients remains weak, while they increase the rates of steroid-induced hyperglycemia. In critically ill patients, hyperglycemia has been associated with worsened prognosis, including increased rates of infection, impaired wound healing, and longer ICU stays [21]. The need for insulin therapy further complicates care, demanding additional monitoring and introducing risks such as hypoglycemia. Hyperglycemia and hypoglycemia are independently associated with increased mortality in critically ill patients [21]. Hence the current clinical practice of routine steroid use in all COPD exacerbations, including the critically ill should be re-evaluated.

An ongoing multicenter, RCT by Ferre et al. (440 patients) comparing methylprednisolone to placebo in ventilated AECOPD will be pivotal in defining the true risk benefit balance [22]. If this trial shows a positive result, future research should be directed towards determining the role of selective steroid therapy in patients with AECOPD. Future trials could also explore standardized steroid dosing and duration, focusing on determining the minimal dose of steroids to reach a clinical benefit, while database research could help us identify treatable traits that benefit from steroid therapy during AECOPD.

### Limitations

Our study findings should be interpreted with caution due to multiple limitations, including small size and number of included RCTs, inclusion of cohort studies with mixed ICU population, and methodological variations between studies. The inclusion of case-control and cohort studies may have introduced confounding bias and contributed to the substantial heterogeneity observed across some key outcomes. Moreover, these studies spanned over a decade during which, ICU practices, ventilator management strategies, and steroid use have evolved, potentially affecting comparability. The criteria for COPD exacerbation diagnosis, ventilation thresholds, steroid regimens and outcome definitions varied between studies. Baseline pulmonary function data (e.g. FEV₁ % predicted or GOLD stage) were often unavailable, reflecting real-world ICU practice but creating potential for confounding and clinical heterogeneity. Not all studies reported outcomes by ventilated subgroup and mode of ventilation, limiting subgroup inference. To mitigate heterogeneity, we performed sensitivity and leave-one-out analyses, detailed in the supplementary material. Two large cohort studies could not be included in several outcome analyses due to lack of outcome specific data, potentially reducing statistical power and introducing selection bias at outcome level.

Despite these limitations, our study provides a focused synthesis of currently available evidence in a clinically important population. Our meta-analysis also supports the current trajectory of clinical guidelines and research for AECOPD which is moving towards a steroid sparing approach.

## Conclusion

The benefit of systemic corticosteroids in AECOPD on ventilation remains unclear. Their use should be weighed against potential adverse effects. Our findings suggest a possible reduction in duration of ventilation in the RCT-only subgroup, without clear benefit for the remaining outcomes, and with significantly increased episodes of hyperglycemia in the systemic steroid group compared with control. Despite several limitations, our study provides a meaningful insight, which could guide direction of future research in this area. Larger, high quality randomized trials are needed to clarify the role and the risk benefit balance of systemic corticosteroids in ventilated AECOPD patients.

## Supplementary Information

Supplementary Material 1. Search strategies. Figure S1: Subgroup analysis of ICU mortality by mode of ventilation. Figure S2: Leave one out analysis for the outcome of length of ICU stay. Figure S3: Leave one out analysis for the outcome of length of duration of ventilation. Figure S4: Cochrane risk of bias assessment for RCTs (RoB 2.0). Table S1: Bias assessment of observational studies (Newcastle-Ottawa scale). Table S2: Definitions.

## Data Availability

All data generated or analyzed during this study are included in this article and its supplementary materials.

## Abbreviations

PICOT: Participants Intervention Control Outcome Type of study
COPD: Chronic obstructive pulmonary disease
AECOPD: Acute exacerbation of chronic obstructive pulmonary disease
ICU: Intensive care unit
MV: Mechanical ventilation or Invasive ventilation
NIV: Non-invasive ventilation
SBT: Spontaneous Breathing Trial
GIB: Gastro-intestinal bleed
VAP: Ventilator associated pneumonia
FEV1: Forced expiratory volume at 1 s
PRISMA: Preferred reporting items for systematic reviews and meta-analysis
NOS: Newcastle-ottawa scale
RCT: Randomized controlled trial
ROB: Risk of bias tool
GOLD: Global strategy for diagnosis, management, and prevention of chronic obstructive lung disease
CHF: Congestive Heart failure
SAPSII: Simplified acute physiology score
APACHEII: Acute physiology and chronic health evaluation
BMI: Body mass index
RR: Risk ratio
OR: Odds ratio
CI: Confidence interval
