# Supplementary material for: Role of systemic corticosteroids in ventilated patients with chronic obstructive pulmonary disease exacerbation: a systematic review and meta-analysis
Source: BMC Pulm Med. 2026 May 18;26:293. doi: 10.1186/s12890-026-04327-7 (PMC13330332; doi:10.1186/s12890-026-04327-7)
Supplement: Supplementary file 1 — Supplementary Material 1. Search strategies. Figure S1: Subgroup analysis of ICU mortality by mode of ventilation. Figure S2: Leave one out analysis for the outcome of length of ICU stay. Figure S3: Leave one out analysis for the outcome of length of duration of ventilation. Figure S4: Cochrane risk of bias assessment for RCTs (RoB 2.0). Table S1: Bias assessment of observational studies (Newcastle-Ottawa scale). Table S2: Definitions. [file 12890_2026_4327_MOESM1_ESM.docx]

**Supplementary Material for “Role of systemic corticosteroids in acute exacerbation of chronic obstructive pulmonary disease on ventilatory support: a systematic review and meta-analysis”**

**Table of contents**

1. Search strategies
2. Tables and figures
   - Figure S1 Subgroup analysis of ICU mortality by mode of ventilation
   - Figure S2 Leave one out analysis for length of ICU stay
   - Figure S3 Leave one out analysis for duration of ventilation
   - Figure S4 Cochrane risk of bias assessment for randomized trials (RoB 2.0)
   - Table S1 Bias assessment of observational studies (Newcastle-Ottawa scale)
   - Table S2 Definitions
3. **Search Strategies**

- **Pubmed**
- ("Chronic Obstructive Pulmonary Diseases" OR COPD OR "Chronic Obstructive Lung Disease" OR "Chronic Obstructive Pulmonary Disease" OR "COAD" OR "Chronic Obstructive Airway Disease" OR "Chronic Airflow Obstruction" OR "Chronic Airflow Obstructions") AND ("systemic steroids" OR "systemic steroid" OR "corticosteroid therapy" OR "systemic corticosteroids" OR "systemic corticosteroid" OR methylprednisolone OR prednisone OR dexamethasone OR prednisolone) AND (exacerbation OR exacerbations OR "acute exacerbation" OR AECOPD OR "acute-on-chronic respiratory failure" OR "respiratory decompensation" OR "acute respiratory deterioration") AND (ICU OR "intensive care" OR "intensive care unit" OR "critical care" OR "critical care unit" OR "intensive therapy" OR "intensive therapy unit" OR "critical illness unit" OR "critically ill" OR "critical care setting" OR "intensive care setting")
- Date: 4/11/2025
- Results: 45
- **Cochrane**
- ("Chronic Obstructive Pulmonary Diseases" OR COPD OR "Chronic Obstructive Lung Disease" OR "Chronic Obstructive Pulmonary Disease" OR "COAD" OR "Chronic Obstructive Airway Disease" OR "Chronic Airflow Obstruction" OR "Chronic Airflow Obstructions") AND ("systemic steroids" OR "systemic steroid" OR "corticosteroid therapy" OR "systemic corticosteroids" OR "systemic corticosteroid" OR methylprednisolone OR prednisone OR dexamethasone OR prednisolone) AND (exacerbation OR exacerbations OR "acute exacerbation" OR AECOPD OR "acute-on-chronic respiratory failure" OR "respiratory decompensation" OR "acute respiratory deterioration") AND (ICU OR "intensive care" OR "intensive care unit" OR "critical care" OR "critical care unit" OR "intensive therapy" OR "intensive therapy unit" OR "critical illness unit" OR "critically ill" OR "critical care setting" OR "intensive care setting")
- Date: 4/11/2025
- Results: 45
- **Embase**
- ("Chronic Obstructive Pulmonary Diseases" OR COPD OR "Chronic Obstructive Lung Disease" OR "Chronic Obstructive Pulmonary Disease" OR "COAD" OR "Chronic Obstructive Airway Disease" OR "Chronic Airflow Obstruction" OR "Chronic Airflow Obstructions") AND ("systemic steroids" OR "systemic steroid" OR "corticosteroid therapy" OR "systemic corticosteroids" OR "systemic corticosteroid" OR methylprednisolone OR prednisone OR dexamethasone OR prednisolone) AND (exacerbation OR exacerbations OR "acute exacerbation" OR AECOPD OR hypoxemia OR hypercapnia OR "acute-on-chronic respiratory failure" OR "acute respiratory failure" OR "respiratory decompensation" OR "acute respiratory deterioration") AND (ICU OR "intensive care" OR "intensive care unit" OR "critical care" OR "critical care unit" OR "intensive therapy" OR "intensive therapy unit" OR "critical illness unit" OR "critically ill" OR "critical care setting" OR "intensive care setting") AND ("mechanical ventilation" OR "non-invasive-positive pressure ventilation" OR NIPPV OR "artificial ventilation" OR "artificially ventilated")
- Date:4/15/2025
- Results:288
- **Citation Search**

- Abd-allah, Hamdy Miligi; Sayed, Wageeh Hassan; El-Monim, Essam Abd. Effect of corticosteroid therapy in patients with an acute exacerbation of chronic obstructive pulmonary disease receiving ventilatory support. Al-Azhar Assiut Medical Journal 19(1):p 175-180, Jan–Mar 2021. | DOI: 10.4103/AZMJ.AZMJ_45_20
- Date: 4/15/2025

1. **Tables and Figures**

**Figure S1:**


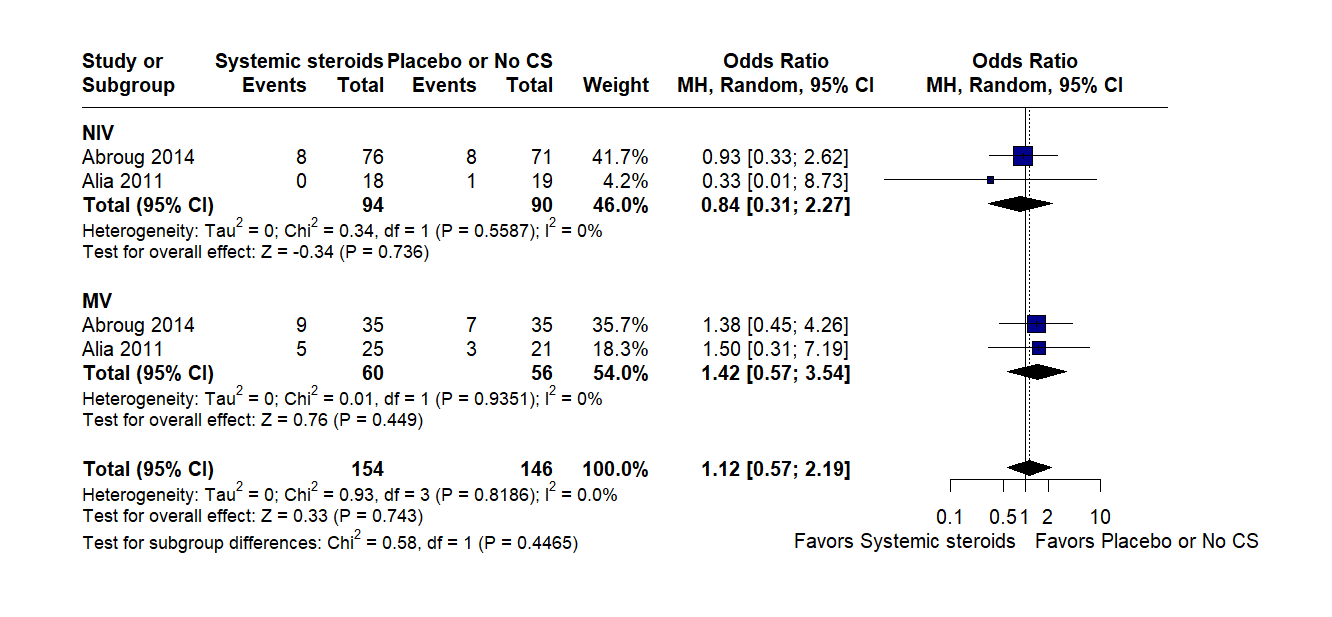


**Figure S2: Leave one out analysis for length of ICU stay**

**
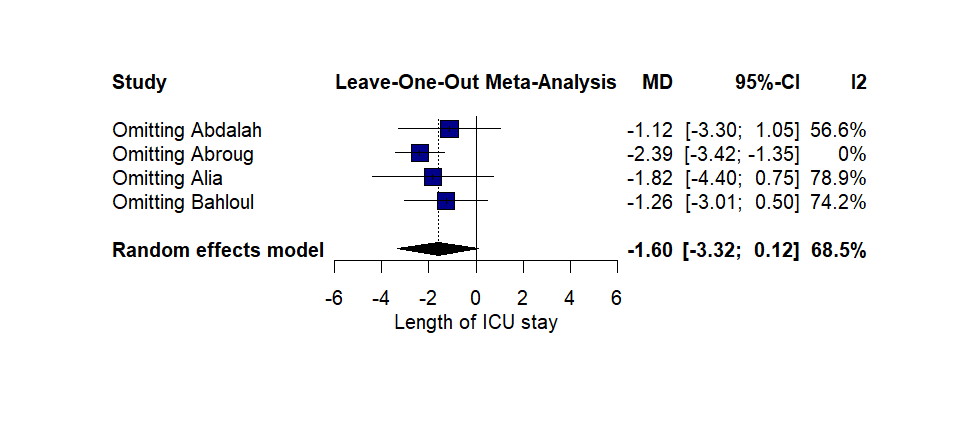
**

**Figure S3: Leave one out analysis for duration of ventilation**

**
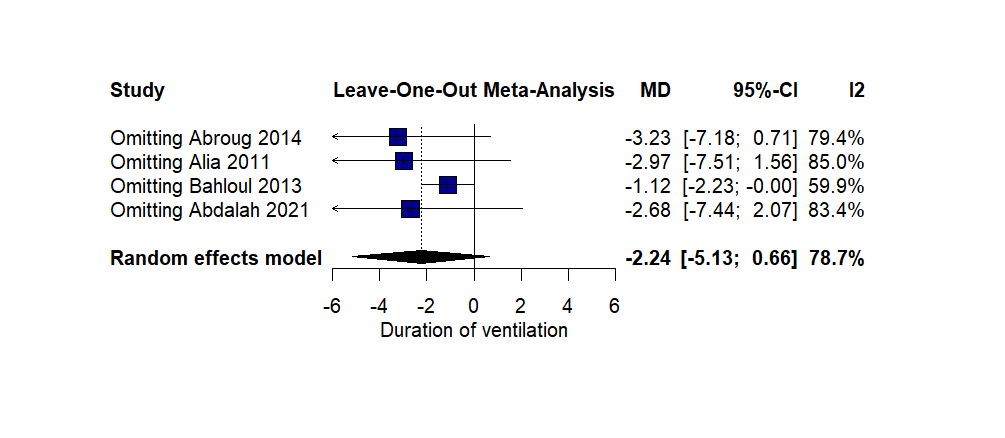
**

**Figure S4: Risk of Bias assessment for Randomized trials (RoB 2.0)**

**
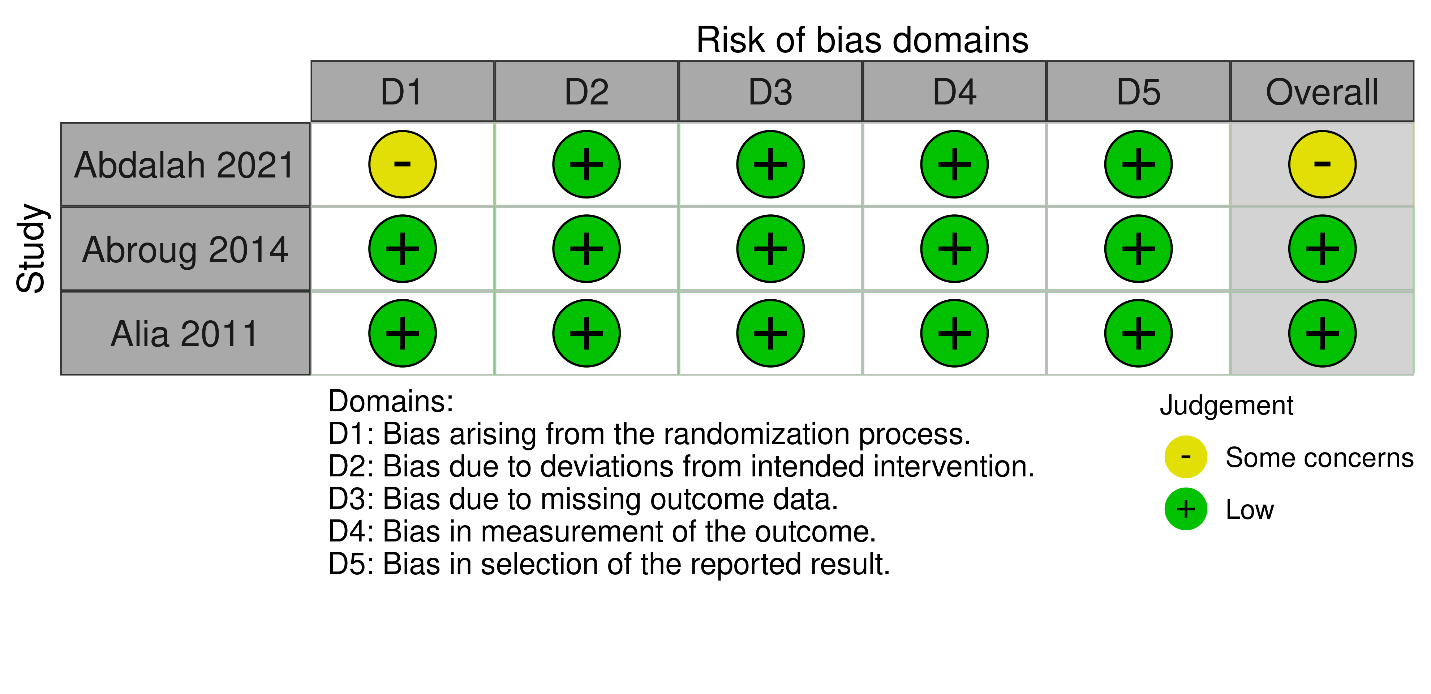
**

**
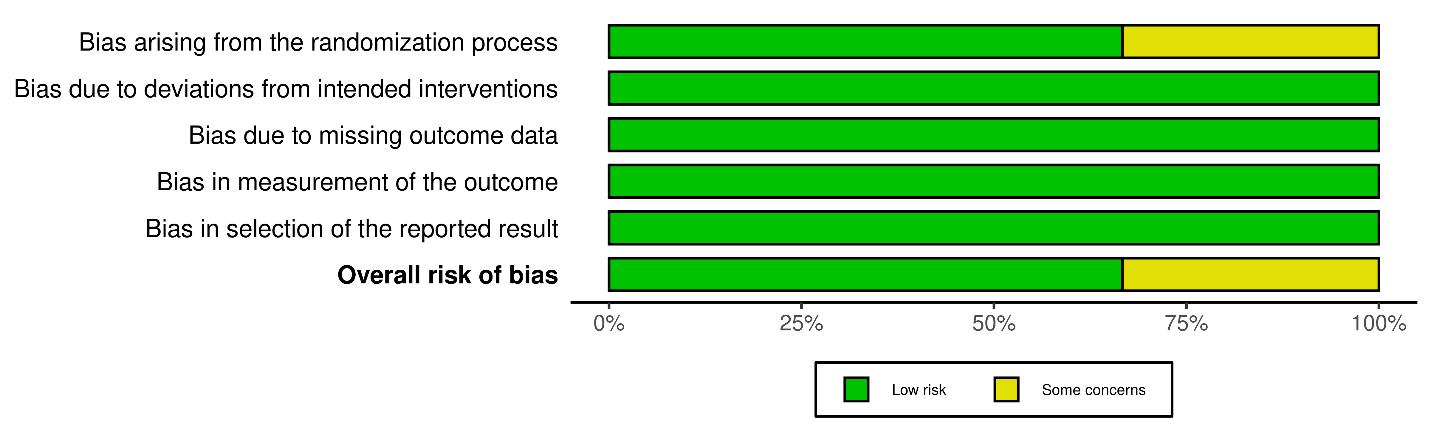
**

**Table S1: Risk of Bias assessment for case control studies**

| **Study** | **Selection** | **Comparability** | **Exposure** |
| --- | --- | --- | --- |
| **Bahloul** | **★★** | **★** | **★★** |
| **Bai** | **★★★** | **★** | **★★** |
| **Galerneau** | **★★★★** | **★** | **★** |

A study can be awarded a maximum of one star for each numbered item following the Newcastle-Ottawa Scale (NOS). In Selection, a study can be awarded a maximum of 4 stars. In Comparability, a study can be awarded a maximum of 1 star. In Exposure, a study can be awarded a maximum of 3 stars. (F.R and T.T)

**Table S2: Definitions**

| **Study** | **Mortality outcome definition** | **Duration of ventilation** | **ICU length of stay** | **NIV failure rate** | **Hyperglycemia** | **Other outcomes/adverse events** |
| --- | --- | --- | --- | --- | --- | --- |
| **Alia et al** | ICU Mortality | Duration of ventilation (for whole population of ventilated patients) and duration of ventilation for NIV and CMV subgroups | Length of ICU stay | In patients treated with NIV, need for intubation if any of the following were present: pH <7.20; pH 7.20–7.25 on 2 measurements 1 hour apart; hypercapnic coma (GCS <8 and PaCO2 ≥60 mmHg); PaO2 <45 mmHg despite maximum tolerated FiO2; and/or cardiac arrest | Initiation of insulin therapy because of blood glucose >120 mg/dL in patients without preexisting diabetes, or increased insulin doses in patients with diabetes | GI bleeding = clinically relevant hematemesis or melena with hemoglobin decrease ≥2 g/dL and no other blood-loss source; arterial hypertension = institution/intensification of antihypertensive therapy because of SBP >160 mmHg and/or DBP >90 mmHg; secondary infection = administration of antibiotics for any proved or suspected infection; hospital-acquired pneumonia = new infiltrate plus ≥2 clinical/microbiologic criteria; ICU-acquired paresis = MRC score <48 |
| **Abroug et al** | ICU Mortality | Length of ventilatory support, defined as the sum of conventional ventilation and NIV in those ventilated with both | Length of ICU stay | In patients initially treated with NIV, failure/intubation if any of the following major criteria present: pH ≤7.20 and below inclusion value; hypercapnic coma (GCS 8 and PaCO2 ≥60 mmHg); PaO2 <45 mmHg despite maximum tolerated inspiratory oxygen fraction; and/or cardiac arrest | Blood glucose ≥180 mg/dL in patients without pre-existing diabetes requiring initiation of insulin therapy, or increase in initial insulin therapy | Significant gastrointestinal bleeding = fall in hemoglobin ≥2 g/dL; NIV success = initial NIV criteria no longer present while breathing without ventilatory assistance for at least 4 hours |
| **Abdalah et al** | Labeled as Mortality outcome (in whole population of ICU ventilated patients) | Reported as duration of mechanical ventilation (whole population of ventilated patients) | Reported as ICU stay | Not clearly defined | Hyperglycemia reported, but no formal threshold or insulin use-based definition clearly stated | Hospital stay; CRP decline reported; ventilator-associated pneumonia and other adverse events mentioned but not formally defined |
| **Bahloul et al** | ICU mortality | Duration of mechanical ventilation (for whole population of ventilated patients) | ICU stay | Not clearly defined | Hyperglycemia ≥8 mmol/L | Complications reported included nosocomial infections, pneumonia, thrombocytopenia, and gastrointestinal bleeding, but detailed formal definitions not provided |
| **Galerneau et al (OUTCOMEREA)** | Primary outcome = composite of death or invasive mechanical ventilation at day 28 after ICU admission; also reported D28 and D90 survival | length of use of NIV (in patients with NIV as ventilatory support) and length of use of IMV (in patients with IMV as ventilatory support) at ICU admission | Length of stay in ICU | Defined as death under NIV or need for IMV for patients treated by NIV in first intention | Hyperglycemia was defined by a fasting blood glucose level >11 mmol/L | Infectious events occurring more than 48 hours after hospitalization in the ICU were considered as nosocomial infectious events. Pneumonia occurring after 48 hours of invasive mechanical ventilation were considered as ventilator-associated pneumonia.  Maximum systolic blood pressure, creatinine blood levels, urea blood levels, fasting blood glucose levels, potassium blood levels, digestive bleeding events, gastric protective agent prescription and digestive endoscopy were also extracted from the database.  Acute kidney injury during ICU Stay (n=1164) was assessed according to the KDIGO clinical practice guidelines. |
| **Le Bai et al (MIMIC IV)** | 28-day mortality; secondary mortality outcomes included ICU mortality and in-hospital mortality | Duration of mechanical ventilation (for whole cohort of ICU patients) Not clear whether sum of NIV + IMV or only IMV | Duration of ICU stay | Not clearly defined | No formal hyperglycemia definition provided | No steroid adverse events reported |
| Abbreviations: ICU, Intensive care unit; NIV, Non-invasive ventilation; IMV, Invasive mechanical ventilation; CMV, conventional mechanical ventilation; CRP, C reactive protein; PaO2, partial pressure of arterial oxygen; FiO2, Fraction of inspired oxygen; MRC, Medical research council score; KDIGO, Kidney Disease: improving Global Outcomes | | | | | | |
